# Association of Medicaid expansion and insurance status, cancer stage, treatment and mortality among patients with cervical cancer

**DOI:** 10.1002/cnr2.1407

**Published:** 2021-05-02

**Authors:** Grace Lee, Edward Christopher Dee, E. John Orav, Daniel W. Kim, Paul L. Nguyen, Alexi A. Wright, Miranda B. Lam

**Affiliations:** ^1^ Harvard Radiation Oncology Program Boston Massachusetts USA; ^2^ Harvard Medical School Boston Massachusetts USA; ^3^ Department of Radiation Oncology Brigham and Women's Hospital/Dana Farber Cancer Institute Boston Massachusetts USA; ^4^ Department of Medicine, Division of General Internal Medicine in Boston Brigham and Women's Hospital Boston Massachusetts USA; ^5^ Department of Biostatistics Harvard T.H. Chan School of Public Health Boston Massachusetts USA; ^6^ Department of Medical Oncology Dana Farber Cancer Institute Boston Massachusetts USA; ^7^ Department of Health Policy and Management Harvard T.H. Chan School of Public Health Boston Massachusetts USA

**Keywords:** cervical cancer, diagnosis, Medicaid expansion, national cancer database, survival, treatment

## Abstract

**Background:**

Currently, little is known about the effect of the Patient Protection and Affordable Care Act's Medicaid expansion on care delivery and outcomes in cervical cancer.

**Aim:**

We evaluated whether Medicaid expansion was associated with changes in insurance status, stage at diagnosis, timely treatment, and survival outcomes in cervical cancer.

**Methods and results:**

Using the National Cancer Database, we performed a difference‐in‐differences (DID) cross‐sectional analysis to compare insurance status, stage at diagnosis, timely treatment, and survival outcomes among cervical cancer patients residing in Medicaid expansion and nonexpansion states before (2011–2013) and after (2014–2015) Medicaid expansion. January 1, 2014 was used as the timepoint for Medicaid expansion. The primary outcomes of interest were insurance status, stage at diagnosis, treatment within 30 and 90 days of diagnosis, and overall survival. Fifteen thousand two hundred sixty‐five patients (median age 50) were included: 42% from Medicaid expansion and 58% from nonexpansion states. Medicaid expansion was significantly associated with increased Medicaid coverage (adjusted DID = 11.0%, 95%CI = 8.2, 13.8, *p* < .01) and decreased rates of uninsured (adjusted DID = −3.0%, 95%CI = −5.2, −0.8, *p* < .01) among patients in expansion states compared with non‐expansion states. However, Medicaid expansion was not associated with any significant changes in cancer stage at diagnosis or timely treatment. There was no significant change in survival from the pre‐ to post‐expansion period in either expansion or nonexpansion states, and no significant differences between the two (DID‐HR = 0.95, 95%CI = 0.83, 1.09, *p* = .48).

**Conclusion:**

Although Medicaid expansion was associated with an increase in Medicaid coverage and decrease in uninsured among patients with cervical cancer, the effects of increased coverage on diagnosis and treatment outcomes may have yet to unfold. Future studies, including longer follow‐up are necessary to understand the effects of Medicaid expansion.

## 1 INTRODUCTION

Tens of millions of United States (US) residents have gained increased access to insurance coverage due to the expansion of Medicaid eligibility that resulted from the Patient Protection and Affordable Care Act (ACA).^1^, ^2^ Prior to ACA, Medicaid eligibility was state‐based but typically included the elderly (age 65+), persons with disabilities, pregnant women, and low‐income families. Post‐ACA, nonelderly adults with incomes ≤138% of the federal poverty line became eligible for Medicaid in states that participate in expansion.^2^ Medicaid expansion has improved the affordability of and access to healthcare, with subsequent lower rates of noninsurance and improvement in outcomes for various conditions.^3^, ^4^, ^5^

Within oncology, Medicaid expansion has been associated with lower rates of noninsurance among nonelderly patients with new cancer diagnoses.^1^, ^6^, ^7^ Researchers have demonstrated increased rates of screening, early‐stage diagnoses, and/or timely treatment for various screening‐amenable malignancies including cervical cancer in expansion states.^1^, ^3^, ^7^, ^8^ Medicaid expansion has also been associated with greater use of cancer surgery among low‐income US residents^9^ as well as diminished racial and socioeconomic disparities in oncologic care access.^8^, ^10^ Given noninsurance predicts poorer outcomes and mortality in cancer patients,^11^, ^12^, ^13^, ^14^, ^15^, ^16^, ^17^, ^18^ Medicaid expansion also has the potential to improve survival in patients with cancer but data is limited. One study of patients with lung cancer found that Medicaid was not associated with improved overall survival compared with noninsurance, suggesting the need for further intervention at the policy level.^19^ A recent study found that in patients with lung, colorectal, and breast cancer had improved survival with Medicaid expansion.^20^

Cervical cancer is a highly screenable cancer in which Medicaid expansion may have a significant impact. There are approximately 126 929 people alive in the US who were diagnosed with cervical cancer from 2001 to 2016. In 2017, 12 831 new cases were reported and 4207 people died of cervical cancer in the US.^21^ Studies have shown that patients who are uninsured, of lower socioeconomic status, or members of racial/ethnic minority groups have lower rates of cervical cancer screening,^22^, ^23^ which is associated with late‐stage disease at diagnosis.^24^, ^25^ Studies conducted prior to the ACA have also demonstrated that noninsurance is associated with increased rates of late‐stage disease at presentation among patients with cervical cancer,^26^ and also partially mediate the increased risk of cervical cancer‐specific mortality in non‐Hispanic Black patients, compared with non‐Hispanic White patients.^27^ Finally, increased time to treatment is associated with worse cervical cancer outcomes.^28^ Currently, there exists limited data on the effect of Medicaid expansion on cervical cancer stage at diagnosis, treatment, and mortality in Medicaid expansion states compared with nonexpansion states.^7^, ^29^, ^30^ Accordingly, the impact of Medicaid expansion on cervical cancer outcomes merits further study.

Given studies suggesting the deleterious effects of noninsurance on patients with cervical cancer,^16^, ^23^, ^24^, ^25^, ^27^ we hypothesized that Medicaid expansion would be associated with improved access to care and screening, and thus earlier stage at diagnosis, greater rates of timely treatment, and improved overall survival in cervical cancer. Using a nationally representative database, we assessed whether Medicaid expansion was associated with changes in cervical cancer outcomes. We examined the interaction between residence in a Medicaid expansion state and diagnosis in the postexpansion period with regards to insurance coverage, stage at diagnosis, timely treatment, and survival.

## 2 METHODS

### 2.1 Data source and patients

Data were obtained from the National Cancer Database (NCDB), a nationwide hospital‐based cancer registry, which captures approximately 70% of new cancer diagnoses in the US from 30% of all US hospitals and contains data on patient, tumor, treatment, and hospital characteristics as well as survival.^31^, ^32^ Our study population included patients (aged 40–64 years) from 2011 to 2015 with newly diagnosed, invasive cervical cancer. Patients <40 years were excluded as NCDB does not report expansion status for this age range. Patients >65 years were excluded as routine screening for cervical cancer is not recommended for these individuals. Those on Medicare were also excluded. Patients diagnosed in 2016 were excluded as survival data were not available for this group. Patients with noninvasive in situ cancers (0.2%) or missing sociodemographic, clinical, geographic, and treatment variables (16%) were excluded. For the analysis of FIGO (International Federation of Gynecology and Obstetrics) stage at diagnosis as the outcome, those with missing/unknown stage were excluded (29%). For the analysis of time to treatment, patients who died <30 days after diagnosis were excluded (0.6%). For analyses with stage at diagnosis as a covariate, an indicator variable was included in the model for missing/unknown stage so that all patients could be included in the analyses. NCDB reports Medicaid expansion status for the state of patient's residence at time of diagnosis as “nonexpansion states,” “January 2014 expansion states,” “early expansion states (2010–2013),” “late expansion states (after January 2014).” We restricted our analyses to include patients from 19 states that expanded Medicaid in January 2014 for the Medicaid expansion states, excluding patients residing in states that expanded Medicaid before (six states) or after (seven states) January 1, 2014, since these patients may dilute the effect of Medicaid expansion that occurred on January 1, 2014. This study was approved by the institutional review board.

### 2.2 Study design

We performed a difference‐in‐differences (DID) cross‐sectional analysis from 2011 to 2015 to compare insurance status, stage at diagnosis, time to treatment, and survival among cervical cancer patients residing in Medicaid expansion and nonexpansion states before (2011–2013) and after (2014–2015) Medicaid expansion. January 1, 2014 was used as the timepoint for Medicaid expansion as it corresponded with the time when 19 states expanded Medicaid.

### 2.3 Independent variables

The primary independent variables of the study were residence in a Medicaid expansion state, diagnosis in the postexpansion period (2014–2015), and the interaction between the two.

### 2.4 Outcomes

The primary outcomes of interest were: (1) insurance status (uninsured, Medicaid), (2) FIGO stage at diagnosis (curable [stage I–III], metastatic [stage IV]), (3) time to initial cancer‐directed treatment (surgery, radiation, or systemic therapy within 30 and 90 days from diagnosis), and (4) overall survival.

### 2.5 Control variables

All models were adjusted for age, Charlson/Deyo comorbidity score,^33^ and urban/rural location.

### 2.6 Statistical analyses

Chi‐square and Kruskal‐Wallis tests were used to compare categorical and continuous variables, respectively. Multivariable linear regression was used to calculate the adjusted DID estimates for insurance status, stage at diagnosis, and timely treatment as a function of residing in a Medicaid expansion state, diagnosis in the postexpansion period, and an interaction between the two; variables included in the model were: age, Charlson/Deyo comorbidity score,^33^ and urban/rural location. Linear models were used based on previous DID studies^1^ and given they provide easily interpretable percentage point estimates of absolute changes.^34^, ^35^ Cox proportional hazard models were used to conduct analogous multivariable analyses of survival since diagnosis. The Kaplan‐Meier method was used to generate survival curves and log‐rank tests were used to compare the survival curves. A two‐sided *p* < .05 was considered as statistically significant. All statistical analyses were performed using Stata/SE 15.1 in May 2020. Four sensitivity analyses were performed (Appendix Methods 2).

## 3 RESULTS

### 3.1 Patient characteristics

A total of 15 265 cervical cancer patients diagnosed between 2011 and 2015 were included in the study. We first compared baseline characteristics of patients in Medicaid expansion (*n* = 6351) and nonexpansion (*n* = 8914) states (Table 1). There were no significant differences in age, histology, FIGO stage, comorbidities, diagnosis year, chemotherapy, or radiation treatment. Compared with those living in nonexpansion states, patients residing in expansion states were less likely to be Black (13% vs. 19%) and uninsured (8% vs. 17%) and were more likely to have Medicaid (31% vs. 23%), a higher median household income ($50 354–63 332 vs. $40227–50 353), receive care at an academic cancer center (53% vs. 42%), have a shorter distance to the hospital facility (11 vs. 14 miles), and have had a hysterectomy (46% vs. 43%).

**TABLE 1:** Baseline patient and treatment characteristics among expansion versus nonexpansion states

Variables	Expansion states *n* = 6351	Nonexpansion states *n* = 8914	*p*‐value
Sociodemographic			
Age (year), median (IQR)	50 (45–56)	50 (45–56)	.16
Race, %			<.01
White	4998 (78.7)	6848 (76.8)	
Black	841 (13.2)	1668 (18.7)	
Asian	342 (5.4)	235 (2.6)	
Other	170 (2.7)	163 (1.8)	
Insurance (%)			<.01
Noninsured	500 (7.9)	1550 (17.4)	
Medicaid	1954 (30.8)	2035 (22.8)	
Other (private, other government)	3897 (61.4)	5329 (59.8)	
Median household income^a^ (%)			<.01
< $40 227	1461 (23.0)	2795 (31.4)	
$40 227–50 353	1451 (22.9)	2439 (27.4)	
$50 354–63 332	1431 (22.5)	1921 (21.6)	
≥ $63 333	2008 (31.6)	1759 (19.7)	
Clinical			
Histology (%)			<.01
Squamous cell carcinoma	4217 (66.4)	6060 (68.0)	
Adenocarcinoma	1824 (28.7)	2368 (26.6)	
Other	310 (4.9)	486 (5.5)	
FIGO stage at diagnosis (%)			.09
I	2319 (36.5)	3255 (36.5)	
II	900 (14.2)	1383 (15.5)	
III	813 (12.8)	1173 (13.2)	
IV	463 (7.3)	606 (6.8)	
Missing/Unknown	1856 (29.2)	2497 (28.0)	
Charlson/Deyo score (%)			.22
0	5382 (84.7)	7497 (84.1)	
1	774 (12.2)	1120 (12.6)	
2	145 (2.3)	199 (2.2)	
≥ 3	50 (0.8)	98 (1.1)	
Geographic			
Facility location (%)			<.01
Atlantic	2312 (36.4)	3944 (44.3)	
Central	2971 (46.8)	2643 (29.7)	
Southwest	66 (1.0)	2100 (23.6)	
Pacific	1002 (15.8)	227 (2.6)	
Rural/Urban (%)			<.01
Urban	6260 (98.6)	8707 (97.7)	
Rural	91 (1.4)	207 (2.3)	
Hospital facility type (%)			<.01
Community cancer program	447 (7.0)	433 (4.9)	
Comprehensive community cancer program	1842 (29.0)	3279 (36.8)	
Academic/research program	3367 (53.0)	3724 (41.8)	
Integrated network cancer program	695 (10.9)	1478 (16.6)	
Distance to hospital (miles), median (IQR)	10.5 (4.7–27.2)	14.0 (6.6–33.4)	<.01
Treatment			
Chemotherapy (%)			.76
Yes	3892 (61.3)	5484 (61.5)	
None	2459 (38.7)	3430 (38.5)	
Radiation (%)			<.01
None	2236 (35.2)	3168 (35.5)	
EBRT alone	1589 (25.0)	2118 (23.8)	
Brachytherapy alone	799 (12.6)	1282 (14.4)	
EBRT + brachytherapy	1727 (27.2)	2346 (26.3)	
Surgery (%)			<.01
None	2938 (46.3)	4351 (48.8)	
Local excision	524 (8.3)	695 (7.8)	
Hysterectomy	2889 (45.5)	3868 (43.4)	
Year of diagnosis (%)			.52
2011	1202 (18.9)	1709 (19.2)	
2012	1281 (20.2)	1716 (19.3)	
2013	1218 (19.2)	1741 (19.5)	
2014	1314 (20.7)	1910 (21.4)	
2015	1336 (21.0)	1838 (20.6)	

Abbreviations: EBRT, external beam radiation therapy; FIGO, International Federation of Gynecology and Obstetrics; IQR, interquartile range.

^a^ Census‐tract level data based on Zip code of residence.

### 3.2 Insurance status

**FIGURE 1:**
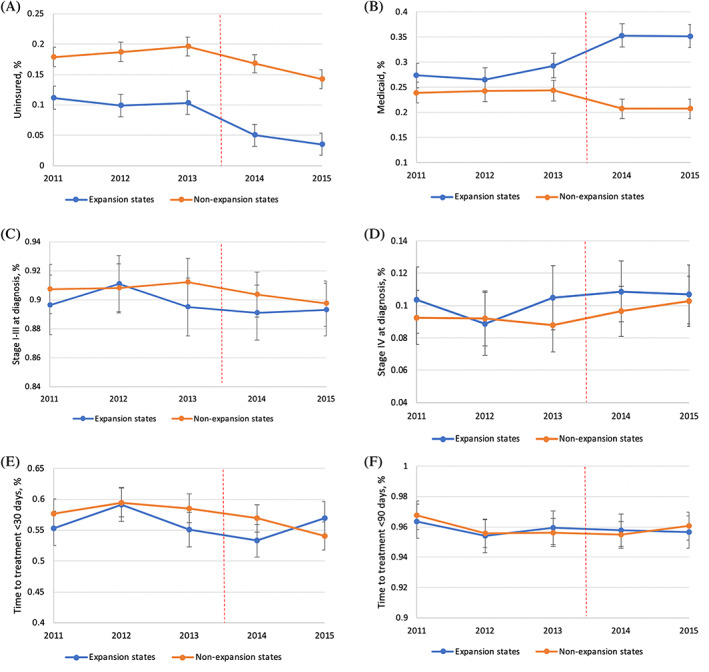
Adjusted Trends in Health Insurance Status (A and B), Cancer Stage at Initial Diagnosis (C and D), and Timely Treatment (E and F) for Cervical Cancer in Medicaid Expansion versus Nonexpansion States: (A) Uninsured, (B) Medicaid, (C) Curable Stage (Stages I–III) Cancer, (D) Metastatic Stage (Stage IV) Cancer, (E) Time to Treatment within 30 days of Diagnosis, (F) Time to Treatment within 90 days of Diagnosis. Participants include patients aged 40–64 years old diagnosed with cervical cancer between January 1, 2012 to December 31, 2015 from the National Cancer Database. Error bars show 95% confidence intervals of estimated margins. The vertical red line represents January 1, 2014, the date of Medicaid expansion

Adjusted trends in insurance status at the time of diagnosis are presented in Figure 1(A) (uninsured) and Figure 1(B) (Medicaid), stratified by Medicaid expansion status. Parallel trends were observed for insurance status in the pre‐ACA period (2011–2013; *p* > .05). The unadjusted difference and adjusted DID for insurance status are shown in Table 2. From the pre‐ to postexpansion period, both expansion (difference = −6.2%, 95%CI = −7.5, −4.9) and nonexpansion (difference = −3.2%, 95%CI −4.8, −1.6) states had a significant decrease in the proportion of uninsured patients. These decreases were significantly different between expansion and nonexpansion states (adjusted DID = −3.0%, 95%CI, −5.2, −0.8). There was a significant increase in the proportion of patients covered by Medicaid in expansion states (difference = 7.5%, 95%CI = 5.2, 9.8) and a significant decrease in the proportion covered by Medicaid in nonexpansion states (difference = −3.3%, 95%CI = −5.1, −1.6) from the pre‐ to postexpansion period. These changes were significantly different (adjusted DID = 11.0%, 95%CI = 8.2, 13.8). Changes in insurance coverage by year in expansion and nonexpansion states are shown in Appendix Table 1.

**TABLE 2:** Changes in insurance status, cancer stage at initial diagnosis, and timely treatment in medicaid expansion versus nonexpansion states

	Expansion states			Nonexpansion states			Adjusted DID (95% CI) and DID *p*‐value
	Before	After	Unadjusted diff (95% CI)	Before	After	Unadjusted diff (95% CI)	
Insurance status							
Uninsured (%)	10.5	4.3	−6.2 (−7.5 to −4.9)	18.7	15.6	−3.2 (−4.8 to −1.6)	−3.0 (−5.2 to −0.8) *p* < .01
Medicaid (%)	27.6	35.1	7.5 (5.2 to 9.8)	24.2	20.9	−3.3 (−5.1 to −1.6)	11.0 (8.2 to 13.8) *p* < .01
Stage at diagnosis							
Stage I–III (%)	90.1	89.2	−0.9 (−2.7 to 0.9)	91.0	90.0	−1.0 (−2.5 to 0.4)	0.0 (−2.3 to 2.3) *p* = .99
Stage IV (%)	9.9	10.8	0.9 (−0.9 to 2.7)	9.0	10.0	1.0 (−0.4 to 2.5)	0.0 (−2.3 to 2.3) *p* = .99
Time from diagnosis to treatment							
≤30 days (%)	56.6	55.0	−1.5 (−4.0 to 1.0)	58.7	55.4	−3.3 (−5.3 to −1.2)	1.6 (−1.6 to 4.8) *p* = .33
≤90 days (%)	95.9	95.7	−0.2 (−1.2 to 0.8)	96.0	95.7	−0.3 (−1.2 to 0.5)	0.1 (−1.2 to 1.4) *p* = .94

Abbreviations: CI, confidence interval; DID, difference‐in‐difference.

### 3.3 Stage at diagnosis

Adjusted trends in FIGO stage at diagnosis are presented in Figure 1(C) (curable, stage I–III) and Figure 1(D) (metastatic, stage IV), stratified by expansion status. Unadjusted difference and adjusted DID for stage at diagnosis are shown in Table 2. Parallel trends were observed for stage at diagnosis in the pre‐ACA period (2011–2013; *p* > .05). Patients diagnosed with curable stage disease nonsignificantly decreased in both expansion and nonexpansion states, and there was no statistically significant difference between the decreases seen in the two groups (adjusted DID = 0.0%, 95%CI = −2.3 to 2.3). The proportion of patients diagnosed with metastatic disease nonsignificantly increased from the pre‐ to postexpansion period in expansion and nonexpansion states, and the increases were not significantly different (adjusted DID = 0.0%, 95%CI = −2.3, 2.3).

### 3.4 Time to treatment

Adjusted trends in timely treatment for cervical cancer are presented in Figure 1(E) (treatment ≤30 days of diagnosis) and Figure 1(F) (treatment ≤90 days of diagnosis), stratified by expansion status. Table 2 summarizes the unadjusted difference and adjusted DID for time to treatment. Parallel trends were observed for treatment within 30 and 90 days in the pre‐ACA period (2011–2013; *p* > .05). There was a slight decrease in the proportion of patients treated ≤30 days in both expansion (difference = −1.5%, 95%CI = −4.0, 1.0) and nonexpansion (difference = −3.3%, 95%CI = −5.3, −1.2) states; however, these decreases were not statistically different between the groups (adjusted DID = 1.6%, 95%CI = −1.6, 4.8). Treatment within 90 days remained similar from the pre‐ to postexpansion periods in both expansion and nonexpansion states, and these changes were not statistically different between the groups (adjusted DID = 0.1%, 95%CI = −1.2, 1.4%).

### 3.5 Overall survival

**FIGURE 2:**
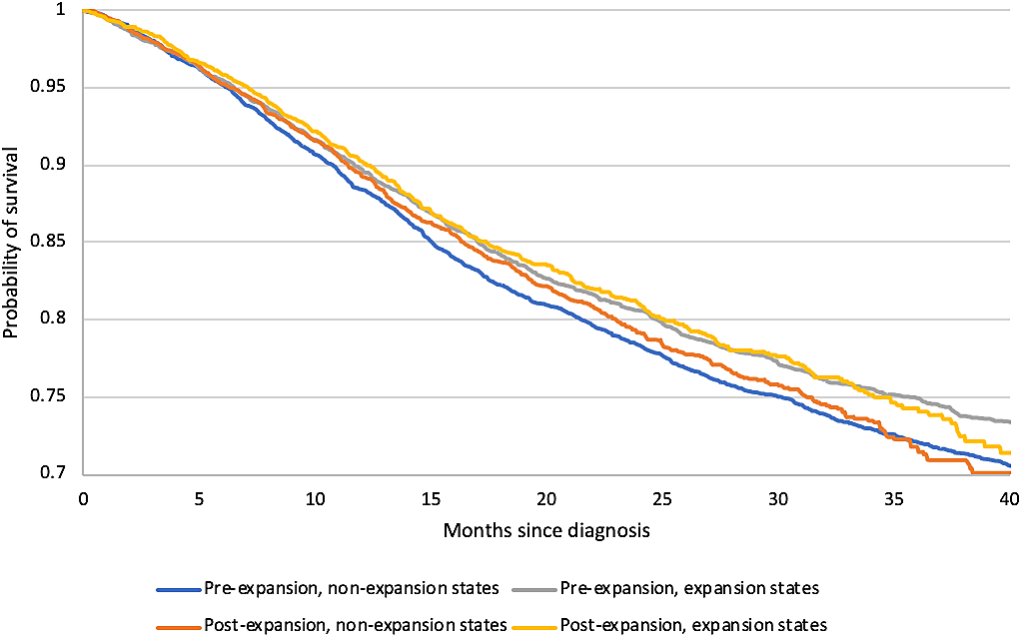
Kaplan–Meier Survival Curves for Medicaid Expansion versus Nonexpansion States in the Pre‐ and Post‐Expansion Period. Participants include patients aged 40–64 years old diagnosed with cervical cancer between January 1, 2012 to December 31, 2015 from the National Cancer Database. There exist statistically significant differences in overall survival between the four curves (log‐rank test *p* = .02) but no significant differences between the pre‐ and postexpansion period curves in expansion (*p* = .72) and nonexpansion (*p* = .53) states

Kaplan‐Meier survival curves (Figure 2; Appendix Figure 1) of expansion and nonexpansion states in the pre‐ and postexpansion periods show statistically significant differences between the four survival curves (*p* < .01) but no significant differences between the pre‐ and postexpansion period curves in both expansion (*p* = .72) and nonexpansion (*p* = .53) states. The adjusted Cox regression model (Table 3) demonstrated no significant change for overall survival in the expansion and nonexpansion states over time. The DID ratio comparing the hazard ratios of death in nonexpansion to expansion states (DID‐HR = 0.95, 95%CI = 0.83, 1.09) indicates no difference in survival between expansion and nonexpansion states. A DID ratio > 1 indicates a greater improvement in expansion versus nonexpansion states, or less worsening in expansion versus nonexpansion states. Results of the four sensitivity analyses are described in Appendix Results 1.

**TABLE 3:** Cox regression of survival in medicaid expansion versus nonexpansion states

	Adjusted	
	Post‐ to pre‐expansion HR (95% CI); *p*‐value	Difference‐in‐difference ratio^a^ (95% CI); *p*‐value
Nonexpansion states	0.96 (0.88–1.05); *p* = .41	Reference
Expansion states	1.01 (0.91–1.13); *p* = .82	0.95 (0.83–1.09); *p* = .48

Abbreviations: CI, confidence interval; HR, hazard ratio.

^a^ Ratio of pre‐ to post‐HR in nonexpansion states compared to pre‐ to post‐HR in combined expansion states. Ratios greater than 1 indicate more improvement in expansion states than in nonexpansion states.

## 4 DISCUSSION

Using a large, nationally representative sample of patients with newly diagnosed cervical cancer, we found that the ACA was associated with significant expansion of Medicaid insurance and decrease in the rate of uninsured patients. However, no significant differences were observed in the stage of cervical cancer at diagnosis, timely treatment, or survival associated with Medicaid expansion.

Our study represents one of few reports investigating the effects of Medicaid expansion on cervical cancer stage at diagnosis, treatment, and mortality. Prior studies have reported mixed results with some demonstrating positive effects of Medicaid expansion on stage at diagnosis and timely treatment.^7^, ^29^, ^30^ Barnes, et al. looked at the impact of early Medicaid expansion in 2010–2011 on outcomes of cervical cancer patients undergoing radiation treatment and found that the six Medicaid expansion states had lower rates of late stage diagnosis (adjusted DID = −5.9%, *p* < .01) compared with nonexpansion states, though without any difference in survival.^29^ Kim, et al. analyzed state cancer registry data from Ohio, a state that expanded Medicaid in 2014, and demonstrated that individuals diagnosed with cervical cancer postexpansion had 37% lower odds of having metastatic disease compared with those diagnosed preexpansion, though the difference was not statistically significant (*p* = .06).^30^ Using quasi‐experimental methods, Albright, et al. recently showed that Medicaid expansion in 2014 was associated with decreased noninsurance rate and increased rate of timely treatment (≤30 days), though without changes in early stage at diagnosis and treatment for cervical cancer.^7^

Given prior studies suggesting the deleterious effects of noninsurance on patients with cervical cancer,^16^, ^23^, ^24^, ^25^, ^27^ we hypothesized that increased Medicaid coverage would be associated with improved access to care and thus increase in earlier stage diagnoses, timely treatment, and better survival in cervical cancer. However, we did not observe such findings. As patients receiving Medicaid often constitute a underserved population, patients receiving Medicaid may be subject to other barriers to care that insurance does not address. For example, Black race, low socioeconomic status, Medicaid payer status, and receipt of care in low‐volume centers (<20 cervical cancer cases per year) have been associated with lower rates of screening^22^, ^23^, ^24^, ^25^ and/or receipt of standard‐of‐care therapy,^36^, ^37^, ^38^, ^39^, ^40^ which have been independently associated with increased late‐stage presentations and cervical cancer‐specific mortality, respectively. Studies have also shown that patients treated at safety‐net hospitals (SNH), defined as facilities with the highest proportion of uninsured or patients receiving Medicaid, receive lower quality care (i.e., lack of concurrent chemoradiation) and a subset of patients (stage II‐III) have worse survival compared to those treated at non‐SNH.^41^ Lack of transportation, which many patients receiving Medicaid may face given lower incomes, has also been associated with late stage cervical cancer diagnosis.^42^ Systemic barriers that affect individual's knowledge regarding how to access health care and navigate the system may further contribute to lack of or delayed screening, and some may not even be aware they are eligible for Medicaid insurance or its benefits.^42^ Accordingly, in addition to Medicaid expansion, policies requiring higher quality care for patients receiving Medicaid as well as policies addressing organizational barriers such as systemic racism/classism are likely necessary for improvement in care access and health outcomes.

Moreover, while studies on other cancers including lung, colon, breast, and other gynecological cancer observed positive effects of Medicaid expansion on cancer diagnosis, treatment, and outcomes,^1^, ^7^, ^8^, ^43^ our study may have not detected significant effects for cervical cancer given underlying differences in cervical cancer and its management compared to other malignancies. For instance, cervical cancer has relatively low incidence (8 out of 100 000 per year) in the United States.^21^ The slow transition from human papillomavirus infection to cervical dysplasia and the development of cervical cancer allows for most cases to be detected early as cervical intraepithelial neoplasia (information not available in NCDB). Further, additional effects of Medicaid expansion on screening and stage at diagnosis may have not been detected given existing publicly sponsored screening programs including the Center for Disease Control's National Breast and Cervical Cancer Early Detection Program (NBCCEDP), which provides low‐income, uninsured, and underserved women access to timely cervical cancer screening and diagnostic services. ACA also eliminated cost sharing in preventive care (including pap smears) for patients with private insurance which may have impacted screening in the privately insured population and counteracted any differential benefit of Medicaid expansion on stage at diagnosis.

The possibility remains that the effect of increased Medicaid coverage on stage at diagnosis, timely treatment, and survival outcomes experienced by patients with cervical cancer has yet to unfold. More follow‐up time may be needed for the positive effects of increased Medicaid to become apparent. Meanwhile, understanding and targeting other disparities that represent barriers to oncologic care will be critical to increase access to care and improve outcomes.

Our study has several limitations. First, retrospective observational data cannot prove causality, despite our use of quasi‐experimental difference‐in‐differences analysis. Second, large databases are subject to errors and heterogeneity in reporting. The suboptimal sensitivity of cancer registry data in correctly identifying insurance status of patients may limit our analyses.^44^ Third, NCDB is not population‐based and does not allow for estimation of population‐based rates of insurance status or cancer stages. Therefore, we are unable to access the previously uninsured individuals who were able become insured through Medicaid expansion, and our analysis is based on individual's state of residence. Moreover, if changes in screening result in a stage shift such that there are more in situ diagnoses that are not included in the cancer registry, then it may appear as an increase in higher stage cancer diagnoses. Fourth, although efforts were made to include important clinical and sociodemographic covariates based on the available data, there are likely other confounders which we could not adjust for that may partially explain some of the disparities identified. For example, we were unable to include state‐level data. However, use of data from hospitals allowed us to examine associations between individual patients and state Medicaid expansion status. Lastly, our findings are limited to patients aged 40‐64 years. Although this range is a limitation of the NCDB dataset, it is possible that trends observed in this age group may extend to younger age groups as well. Future studies may examine these trends in other age groups compared with those presented here.

Overall, our study demonstrated that although Medicaid expansion was associated with a significant increase in Medicaid coverage among patients with cervical cancer, it may be too early to see the downstream effects of this expansion on diagnosis, treatment, and survival outcomes. Our findings also underscore the multifactorial nature of disparities in care. Patients covered by Medicaid may experience barriers to care that precede the need for care that Medicaid facilitates. Further assessment of the roots of these disparities may lead to an increased understanding of ways in which these disparities can be mitigated.

## CONFLICT OF INTEREST

The authors have stated explicitly that there are no conflicts of interest in connection with this article.

## AUTHOR CONTRIBUTIONS

All authors had full access to the data in the study and take responsibility for the integrity of the data and the accuracy of the data analysis. *Conceptualization*, G.L., E.C.D., M.B.L.; *Methodology*, G.L., E.C.D., E.J.O., D.W.K., A.A.W., M.B.L.; *Investigation*, M.B.L.; *Formal Analysis*, G.L., E.C.D., E.J.O.; *Resources*, E.J.O., D.W.K., P.L.N., A.A.W., M.B.L.; *Writing ‐ Original Draft*, G.L., E.C.D., M.B.L.; *Writing ‐ Review & Editing*, G.L., E.C.D., E.J.O., D.W.K., P.L.N., A.A.W., M.B.L.; *Visualization*, G.L.; *Supervision*, M.B.L.; *Funding Acquisition*, M.B.L.; *Software*, G.L., E.C.D.; *Validation*, E.J.O., D.W.K., P.L.N., A.A.W., M.B.L.; *Data Curation*, M.B.L.; *Project Administration*, M.B.L.

## ETHICAL STATEMENT

This study was approved by the institutional review board.

## Data Availability

The primary dataset (National Cancer Database) used for this study is publicly available through the American College of Surgeons (https://www.facs.org/quality-programs/cancer/ncdb). The specific datasets included in this study are available on reasonable request from the corresponding author.
