# Supplementary material for: Association of Medicaid expansion and insurance status, cancer stage, treatment and mortality among patients with cervical cancer
Source: Cancer Rep (Hoboken). 2021 May 2;4(6):e1407. doi: 10.1002/cnr2.1407 (PMC8714536; doi:10.1002/cnr2.1407)
Supplement: Supplementary file 1 [file CNR2-4-e1407-s001.docx]

**Appendix**

**Appendix Methods 1.**

Patients with newly diagnosed cervical cancer were identified in NCDB by International Classification of Diseases for Oncology, 3rd Edition [ICD-O-3] codes C53.0-C53.1, C53.8-C53.9.

**Appendix Methods 2.**

*Sensitivity Analyses*

Four sensitivity analyses were performed. First, we expanded our analyses to include patients from states that expanded Medicaid before (6 states) or after (7 states) January 1, 2014 for the Medicaid expansion states. Second, we restricted our analyses to low-income patients with median household income in the bottom quartile (<$40,227); the cancer registry data estimates median household income for each patient’s area of residence by matching the zip code of the patient recorded at the time of diagnosis against files derived from the 2016 American Community Survey Data. Third, analyses that examined associations between Medicaid expansion and insurance status, FIGO stage at diagnosis, and time to treatment were repeated with the addition of patients who were diagnosed in 2016 (excluded in the main analyses due to lack of survival data), given the effect of Medicaid expansion may be delayed and not evident within two years. Fourth, propensity score weighted analyses were conducted for the difference-in-difference analyses for changes in insurance status, cancer stage at initial diagnosis, and timely treatment in Medicaid expansion vs. non-expansion states; the following variables included in the adjusted models were weighted: age, Charlson/Deyo comorbidity score,^25^ and urban/rural location.

**Appendix Results 1.**

*Sensitivity Analyses*

The results of the sensitivity analyses conducted with 1) inclusion of early and late Medicaid expanders, 2) exclusion of patients with median household income >$40,227, 3) inclusion of patient data from 2016, 4) propensity score weighting are summarized in **Appendix Tables 2-7** and **Appendix Figure 2**. First, analysis including early and late Medicaid expanders revealed significant increase in patients receiving Medicaid but no significant difference in uninsured patients from the pre- to post-expansion period between expansion and non-expansion states, which is in contrary to our main analyses which showed a significant decrease in uninsured patients. There was no significant change in the direction or significance of effect estimates for stage at diagnosis and timely treatment. There was no significant change in the overall survival differences between the groups. Second, analysis restricted to low-income patients revealed significant increase in patients receiving Medicaid and decrease in uninsured patients from the pre- to post-expansion period between expansion and non-expansion states, similar to findings of our main analyses but with greater effect. There was no significant change in the direction or significance of effect estimates for stage at diagnosis and timely treatment. There was no significant change in the overall survival differences between the groups. Third, analyses conducted including patients diagnosed in 2016 demonstrated significant increase in patients receiving Medicaid but no significant difference in uninsured patients from the pre- to post-expansion period between expansion and non-expansion states, which is in contrary to our main analyses which showed a significant decrease in uninsured patients. There were no significant differences in stage at diagnosis or treatment ≤90 days. However, there was a significant difference in the proportion of patients who received treatment ≤30 days; there was a decrease in the proportion of patients treated ≤30 days in both expansion and non-expansion states and the decrease in the expansion states was significantly lower (adjusted DID=3.2%, 95%CI=0.3, 6.1). Fourth, analyses conducted with propensity score weighting showed significant increase in patients receiving Medicaid and decrease in uninsured patients from the pre- to post-expansion period between expansion and non-expansion states, similar to findings of our main analyses. There were no significant differences in stage at diagnosis and timely treatment.

**Appendix Table 1.** Changes in Insurance Status in Medicaid Expansion vs. Non-expansion States over 2011-2016

|  | Uninsured | Medicaid | Private | Other government |
| --- | --- | --- | --- | --- |
| Expansion states |  |  |  |  |
| 2011 | 134 (11.2) | 326 (27.1) | 732 (60.9) | 10 (0.8) |
| 2012 | 127 (9.9) | 340 (26.5) | 798 (62.3) | 16 (1.3) |
| 2013 | 126 (10.3) | 357 (29.3) | 726 (59.6) | 9 (0.7) |
| 2014 | 66 (5.0) | 463 (35.2) | 776 (59.1) | 9 (0.7) |
| 2015 | 47 (3.5) | 468 (35.0) | 810 (60.6) | 11 (0.8) |
| 2016 | 31 (2.5) | 404 (32.6) | 792 (63.9) | 12 (1.0) |
| Non-expansion states |  |  |  |  |
| 2011 | 305 (17.9) | 410 (24.0) | 952 (55.7) | 42 (2.5) |
| 2012 | 321 (18.7) | 417 (24.3) | 938 (54.7) | 40 (2.3) |
| 2013 | 341 (19.6) | 425 (24.4) | 928 (53.3) | 47 (2.7) |
| 2014 | 32 (16.8) | 397 (20.8) | 1,144 (59.9) | 48 (2.5) |
| 2015 | 262 (14.3) | 386 (21.0) | 1,136 (61.8) | 54 (2.9) |
| 2016 | 273 (15.1) | 368 (20.4) | 1,122 (62.2) | 42 (2.3) |

**Appendix Table 2**. Changes in Insurance Status, Cancer Stage at Initial Diagnosis, and Timely Treatment in Medicaid Expansion vs. Non-expansion States Including States that Expanded Before or After 2014

|  | Expansion states | | |  | Non-expansion states | | | Adjusted DID (95% CI); DID P-value |  |  |  |
| --- | --- | --- | --- | --- | --- | --- | --- | --- | --- | --- | --- |
|  | Before | After | Unadjusted diff  (95% CI) |  | Before | After | Unadjusted diff  (95% CI) |  |  |  |  |
| Insurance Status | |  |  |  |  |  |  |  |  |  |  |
| Uninsured, % | 9.0 | 4.9 | -4.1 (-5.0 to -3.2) |  | 18.7 | 15.6 | -3.2 (-4.8 to -1.6) | -0.9 (-2.6 to 0.8)  p=0.30 |  |  |  |
| Medicaid, % | 29.1 | 32.3 | 3.2 (1.6 to 4.8) |  | 24.2 | 20.9 | -3.3 (-5.1 to -1.6) | 6.5 (4.1 to 9.0)  p<0.01 |  |  |  |
| Stage at Diagnosis | |  |  |  |  |  |  |  |  |  |  |
| Stage I-III, % | 90.4 | 89.7 | -0.6 (-2.0 to 0.6) |  | 91.0 | 90.0 | -1.0 (-2.5 to 0.4) | 0.3 (-1.6 to 2.2)  p=0.73 |  |  |  |
| Stage IV, % | 9.6 | 10.3 | 0.6 (-0.6 to 2.0) |  | 9.0 | 10.0 | 1.0 (-0.4 to 2.5) | -0.3 (-2.2 to 1.6) p=0.73 |  |  |  |
| Time from Diagnosis to Treatment | | | |  |  |  |  |  |  |  |  |
| ≤30 days, % | 55.9 | 53.4 | -2.4 (-4.2 to -0.7) |  | 58.7 | 55.4 | -3.3 (-5.3 to -1.2) | 0.8 (-1.9 to 3.5)  p=0.58 |  |  |  |
| ≤90 days, % | 94.9 | 94.4 | -0.5 (-1.3 to 0.3) |  | 96.0 | 95.7 | -0.3 (-1.2 to 0.5) | -0.2 (-1.4 to 1.0) p=0.74 |  |  |  |

*Abbreviations:* CI, confidence interval; DID, difference-in-difference

**Appendix Table 3.** Cox Regression of Survival in Medicaid Expansion vs. Non-expansion States Including States that Expanded Before or After 2014

|  | **Post- to Pre-expansion HR**  **(95% CI); P-value** | **Difference-in-Difference ratio***  **(95% CI); P-value** |
| --- | --- | --- |
|  | | |
| **Non-expansion states** | 0.97 (0.89-1.06); p=0.50 | Reference |
| **Expansion states** | 0.98 (0.91-1.06); p=0.61 | 0.99 (0.88-1.11); p=0.86 |

*Ratio of pre- to post- HR in non-expansion states compared to pre- to post- HR in combined expansion states. Ratios greater than 1 indicate more improvement in expansion states than in non-expansion states.

*Abbreviations*: HR, hazard ratio; CI, confidence interval

**Appendix Table 4.** Changes in Insurance Status, Cancer Stage at Initial Diagnosis, and Timely Treatment in Medicaid Expansion vs. Non-expansion States for Low-income Patients

|  | Expansion states | | |  | Non-expansion states | | | Adjusted DID (95% CI); DID P-value |  |  |  |
| --- | --- | --- | --- | --- | --- | --- | --- | --- | --- | --- | --- |
|  | Before | After | Unadjusted diff  (95% CI) |  | Before | After | Unadjusted diff  (95% CI) |  |  |  |  |
| Insurance Status | |  |  |  |  |  |  |  |  |  |  |
| Uninsured, % | 14.6 | 4.7 | -9.8 (-13.0 to -6.7) |  | 22.5 | 21.7 | -0.8 (-3.9 to 2.3) | -9.1 (-13.9 to -4.2) p<0.01 |  |  |  |
| Medicaid, % | 40.6 | 51.5 | 10.9 (5.7 to 16.0) |  | 33.3 | 27.4 | -5.9 (-9.3 to -2.4) | 16.9 (10.9 to 22.9) p<0.01 |  |  |  |
| Stage at Diagnosis | |  |  |  |  |  |  |  |  |  |  |
| Stage I-III, % | 89.3 | 85.8 | -3.4 (-7.4 to 0.6) |  | 89.8 | 89.5 | -0.4 (-3.0 to 2.3) | -3.2 (-7.9 to 1.5) p=0.19 |  |  |  |
| Stage IV, % | 10.7 | 14.2 | 3.4 (-0.6 to 7.4) |  | 10.2 | 10.5 | 0.4 (-2.3 to 3.0) | 3.2 (-1.5 to 7.9) p=0.19 |  |  |  |
| Time from Diagnosis to Treatment | | | |  |  |  |  |  |  |  |  |
| ≤30 days, % | 54.9 | 52.5 | -2.4 (-7.6 to 2.8) |  | 52.4 | 48.5 | -3.9 (-7.6 to -0.1) | 1.5 (-4.9 to 7.9) p=0.65 |  |  |  |
| ≤90 days, % | 94.7 | 95.6 | 0.8 (-1.4 to 3.1) |  | 94.0 | 93.9 | -0.2 (-2.0 to 1.6) | 1.0 (-2.0 to 4.0) p=0.51 |  |  |  |

*Abbreviations:* CI, confidence interval; DID, difference-in-difference

**Appendix Table 5.** Cox Regression of Survival in Medicaid Expansion vs. Non-expansion States for Low-income Patients

|  | **Post- to Pre-expansion HR**  **(95% CI); P-value** | **Difference-in-Difference ratio***  **(95% CI); P-value** |
| --- | --- | --- |
|  | | |
| **Non-expansion states** | 0.93 (0.80-1.08); p=0.35 | Reference |
| **Expansion states** | 1.11 (0.91-1.36); p=0.29 | 0.84 (0.65-1.07); p=0.15 |
|  |  |  |

*Ratio of pre- to post- HR in non-expansion states compared to pre- to post- HR in combined expansion states. Ratios greater than 1 indicate more improvement in expansion states than in non-expansion states.

*Abbreviations*: HR, hazard ratio; CI, confidence interval

**Appendix Table 6.** Changes in Insurance Status, Cancer Stage at Initial Diagnosis, and Timely Treatment in Medicaid Expansion vs. Non-expansion States for the 2011-2016 Cohort

|  | Expansion states | | |  | Non-expansion states | | | Adjusted DID (95% CI) and DID P-value |  |  |
| --- | --- | --- | --- | --- | --- | --- | --- | --- | --- | --- |
|  | Before | After | Unadjusted diff  (95% CI) |  | Before | After | Unadjusted diff  (95% CI) |  |  |  |
| Insurance Status | |  |  |  |  |  |  |  |  |  |
| Uninsured, % | 10.5 | 3.7 | -6.8 (-7.9 to -5.6) |  | 18.7 | 15.4 | -3.3 (-4.7 to -1.9) | -1.2 (-2.7 to 0.3)  p=0.13 |  |  |
| Medicaid, % | 27.6 | 34.3 | 6.7 (4.6 to 8.8) |  | 24.2 | 20.7 | -3.5 (-5.1 to -1.9) | 6.4 (4.2 to 8.6)  p<0.01 |  |  |
| Stage at Diagnosis | |  |  |  |  |  |  |  |  |  |
| Stage I-III, % | 90.1 | 88.8 | -1.3 (-2.9 to 0.3) |  | 91.0 | 89.7 | -1.3 (-2.6 to 0.0) | -0.1 (-2.2 to 2.0)  p=0.92 |  |  |
| Stage IV, % | 9.9 | 11.2 | 1.3 (-0.3 to 2.9) |  | 9.0 | 10.3 | 1.3 (0.0 to 2.6) | 0.1 (-2.0 to 2.2)  p=0.92 |  |  |
| Time from Diagnosis to Treatment | | | |  |  |  |  |  |  |  |
| ≤30 days, % | 56.6 | 55.7 | -0.9 (-3.1 to 1.3) |  | 58.7 | 54.5 | -4.2 (-6.1 to -2.3) | 3.2 (0.3 to 6.1)  p=0.03 |  |  |
| ≤90 days, % | 95.9 | 95.7 | -0.2 (-1.1 to 0.7) |  | 96.0 | 95.6 | -0.4 (-1.2 to 0.3) | 0.3 (-1.0 to 1.4)  p=0.73 |  |  |

*Abbreviations:* CI, confidence interval; DID, difference-in-difference

**Appendix Table 7**. Propensity Score Weighted Difference-in-Difference Analysis for Changes in Insurance Status, Cancer Stage at Initial Diagnosis, and Timely Treatment in Medicaid Expansion vs. Non-expansion States

|  | Adjusted DID (95% CI)  and DID P-value |
| --- | --- |
|  |  |
| Insurance Status | |
| Uninsured, % | -3.1 (-5.1 to -1.1); p<0.01 |
| Medicaid, % | 11.1 (8.2 to 13.9); p<0.01 |
| Stage at Diagnosis | |
| Stage I-III, % | 0.0 (-2.3 to 2.3); p=0.97 |
| Stage IV, % | 0.0 (-2.3 to 2.3); p=0.97 |
| Time from Diagnosis to Treatment | |
| ≤30 days, % | 1.6 (-1.7 to 4.8); p=0.34 |
| ≤90 days, % | 0.1 (-1.3 to 1.4); p=0.94 |

*Abbreviations:* CI, confidence interval; DID, difference-in-difference

**Appendix Figure 1.** Kaplan Meier Survival Curves for A. Medicaid Expansion and B. Non-expansion States


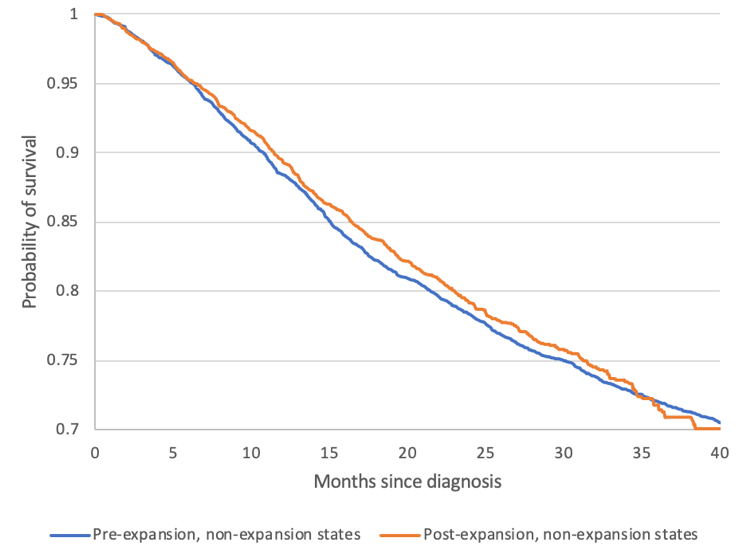
**
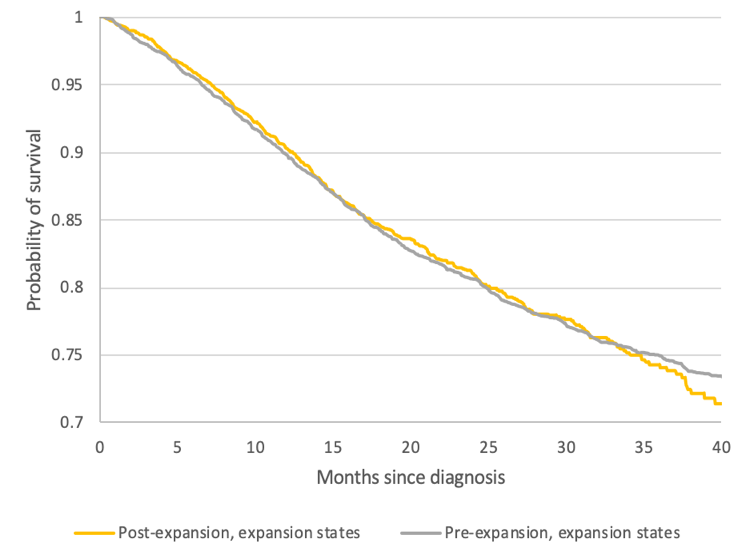
**A. Expansion State B. Non-expansion State

.

*log-rank test p=0.72 *log-rank test p=0.53

**Appendix Figure 2.** Patterns in Health Insurance Status (A-B), Cancer Stage at Initial Diagnosis (C-D), and Timely Treatment (E-F) in Medicaid Expansion vs. Non-expansion States for the 2011-2016 Cohort

**A.** Uninsured

**
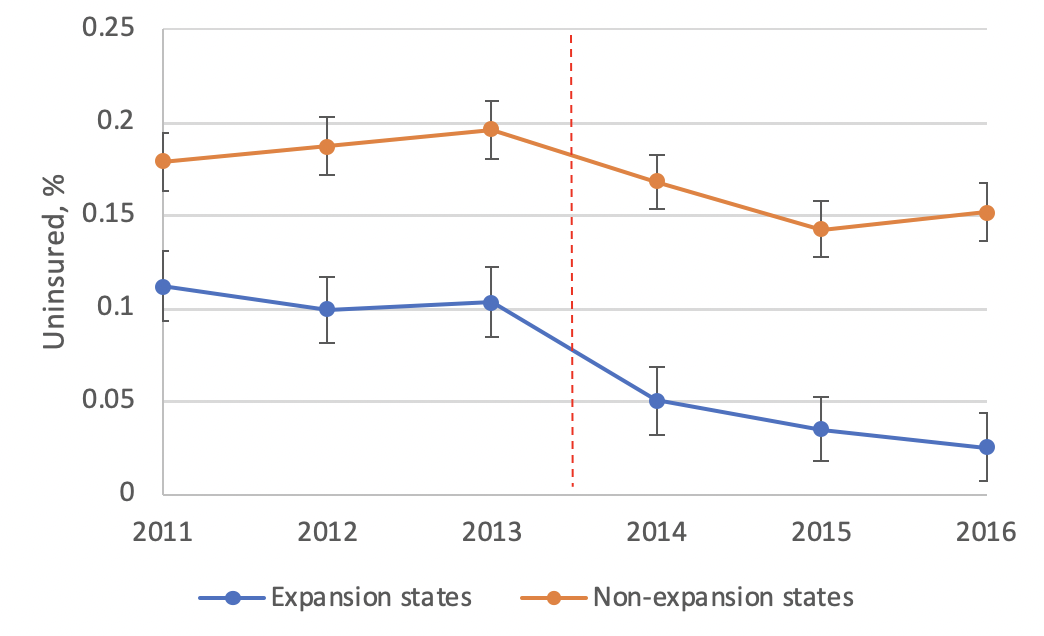
**

**B.** Medicaid


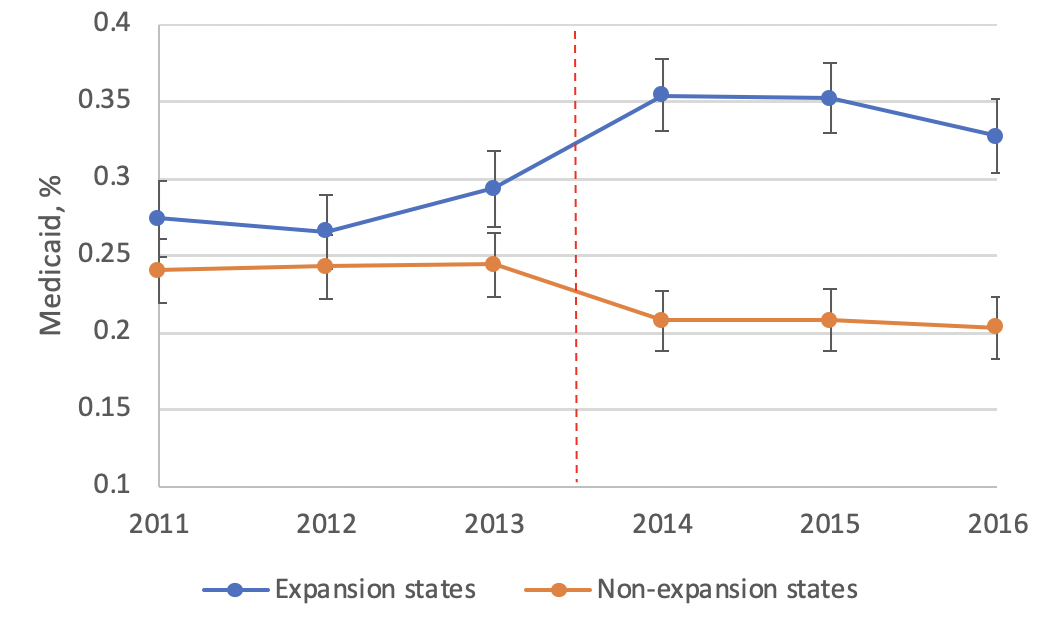


**C.** Curable Stage (Stage I-III) Cancer


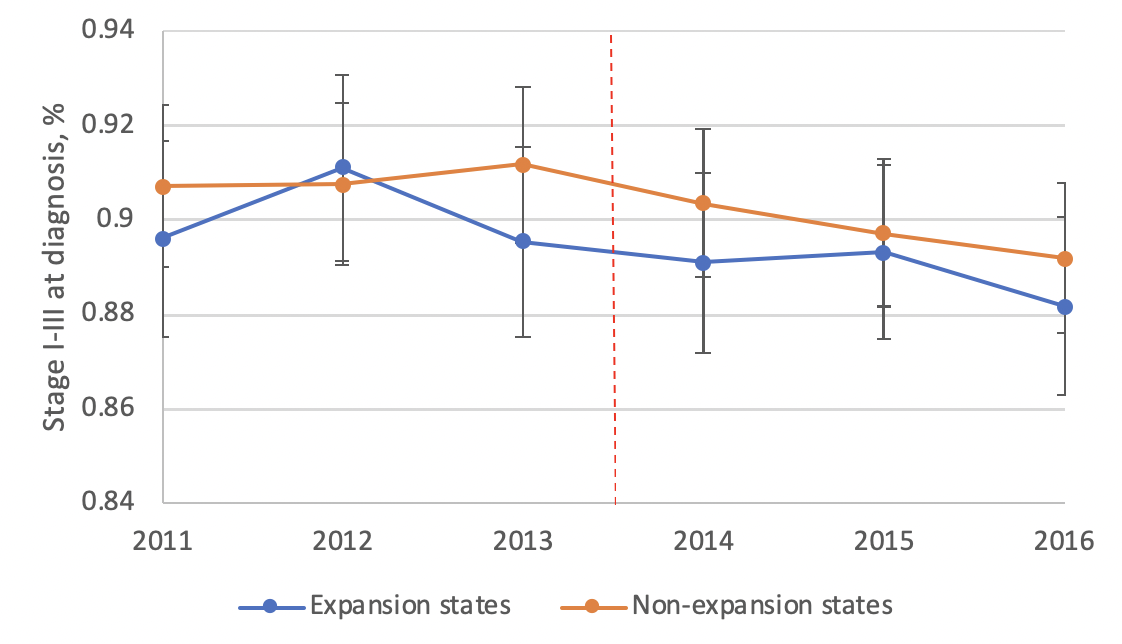


**D.** Metastatic Stage (Stage IV) Cancer


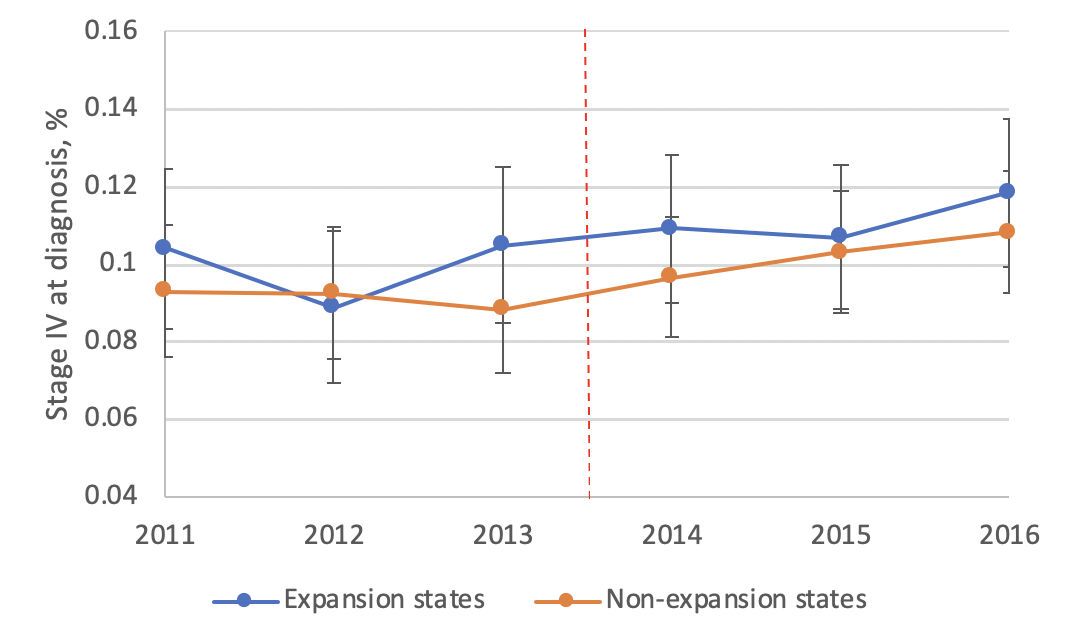


**E.** Time to Treatment within 30 days of Diagnosis


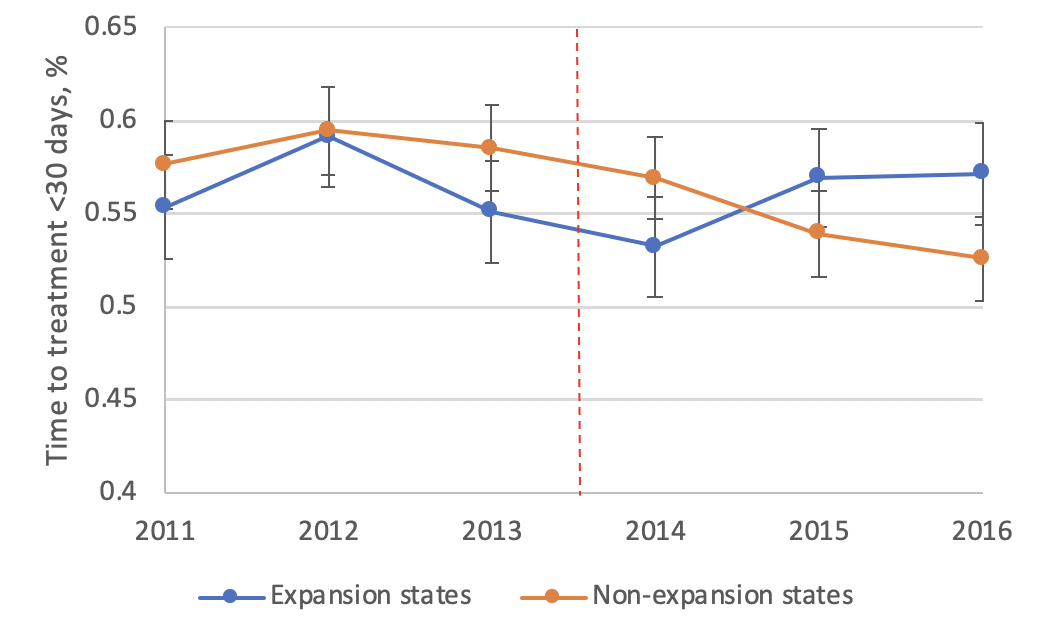


**F.** Time to Treatment within 90 days of Diagnosis

**
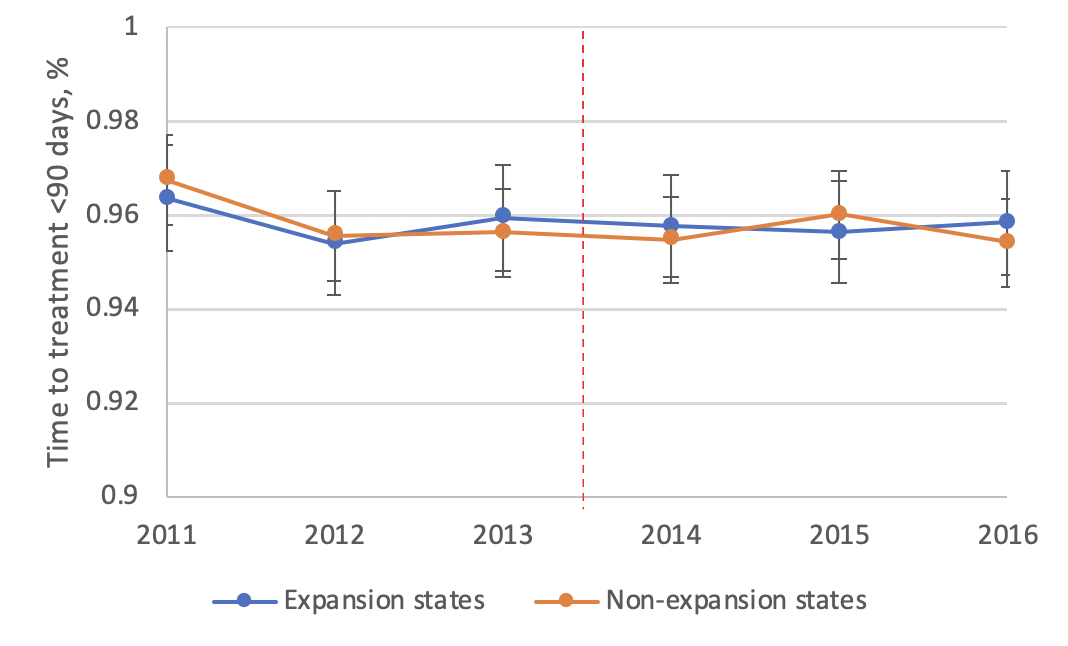
**
